# *Coccidioides immitis* soft tissue infection mimicking pseudofolliculitis barbae

**DOI:** 10.1002/ccr3.1421

**Published:** 2018-02-21

**Authors:** Wendy Gerstein, Valeria Ilieva

**Affiliations:** ^1^ Department of Internal Medicine New Mexico VA Health Care Service/University of New Mexico 1501 San Pedro Ave, SE Albuquerque New Mexico 87108; ^2^ Division of Infectious Diseases NMVAHCS/University of New Mexico 1501 San Pedro Ave, SE Albuquerque New Mexico 87108

**Keywords:** Dermatology, fungal infection, pseudofolliculitis, skin biopsy, skin infections

## Abstract

Endemic fungal infections can present atypically and should be considered in the differential diagnosis of any soft tissue infection not responding appropriately to antibiotic therapy. Diagnosis can be confirmed with a biopsy. Most fungal soft tissue infections require extended duration of treatment.

Patient is a 61‐year‐old male from southern New Mexico who presented with complaints of swelling, itching, and pain around his beard. He was diagnosed with pseudofolliculitis barbae and prescribed antibiotics. He returned 6 months later with worsening symptoms and a new complaint of his facial hair falling out in clumps. Antibiotics were prescribed again, and he was referred to a dermatologist who continued antibiotics plus added oral steroids. The symptoms worsened including facial disfigurement (Fig. 1). Skin biopsy showed *Coccidioides species* (Fig. 2 {PAS stain of spherule}, Fig. 3 {arthroconidia}, white arrows). Laboratories revealed a normal WBC with 22% eosinophils and serum cocci IgG 0.841 and IgM 0.208 (reference 0.0–0.149) with CF titer of 1:4. Computed tomography scan of the jaw confirmed deep soft tissue involvement. *C. immitis* is an endemic fungal infection found in the southwest United States, but usually presents as a self‐limited respiratory illness due to inhalation of arthroconidia. *C. immitis* skin infections are usually due to dissemination from a respiratory infection but there are case reports of cutaneous inoculations, which may have played a role in this patient's presentation as he had no history nor evidence for respiratory involvement 1. Patient was treated with oral fluconazole 800 mg daily with significant improvement at 2 months (Fig. 4). He completed 12 months of therapy with complete resolution and no complications. This case is a reminder to consider fungal etiologies for persistent or resistant skin infections.

**Figure 1:**
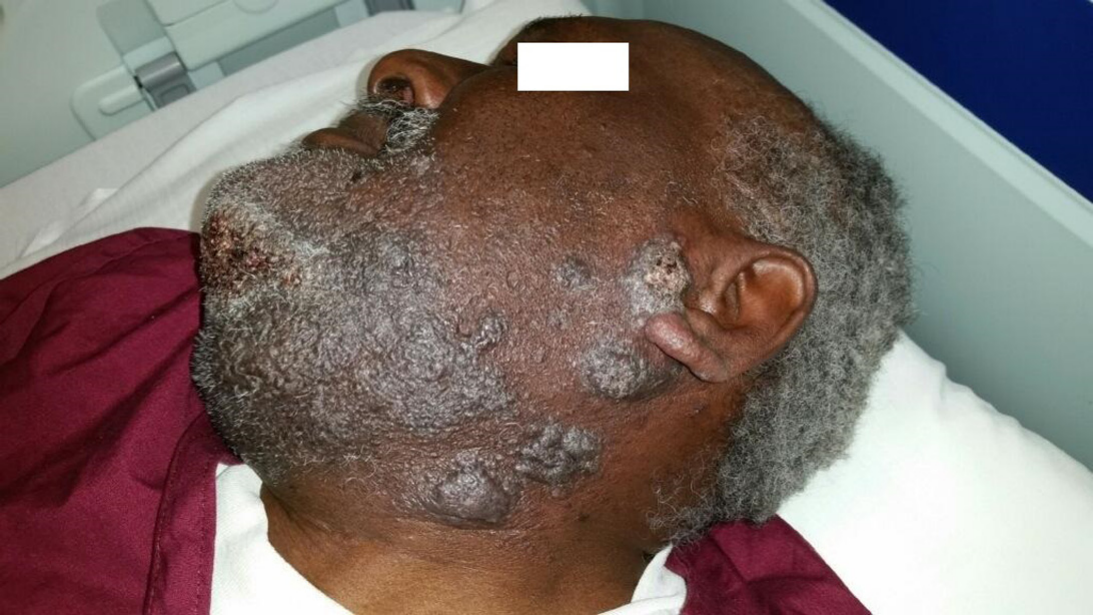
Facial disfigurement from *coccidioides* infection.

**Figure 2:**
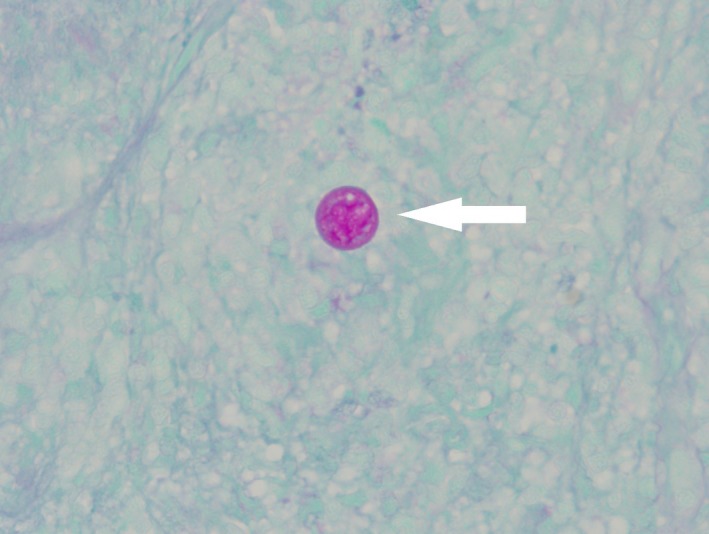
PAS stain of *coccidioides* spherules.

**Figure 3:**
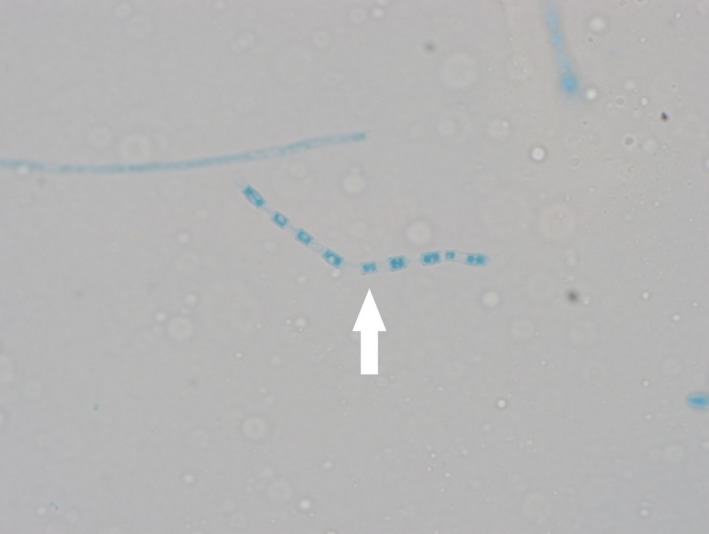
*Coccidioides* arthroconidia.

**Figure 4:**
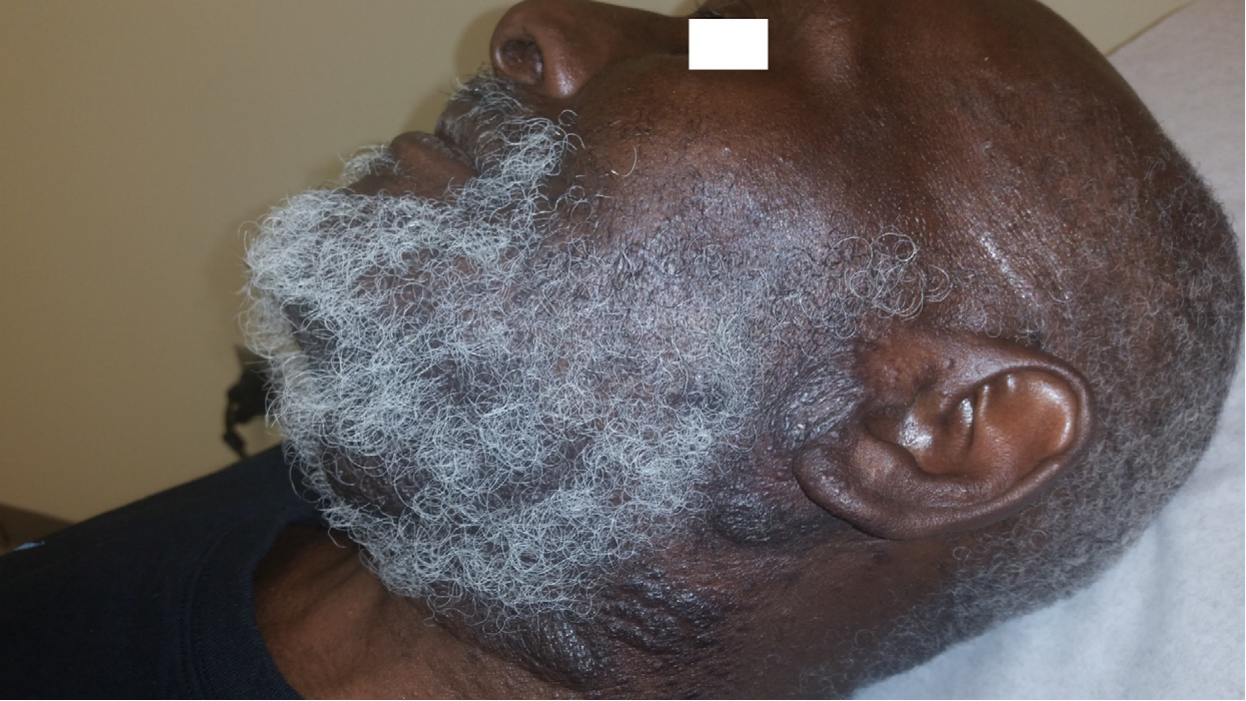
Two months into treatment.

## Conflict of Interest

None declared.

## Authorship

WG: contributed to patient care, drafted the manuscript, and obtained the patient photographs. VI: contributed to patient care, obtained photographs of the organism, and revised the manuscript.
